# Targeted Deletion of Neuropeptide Y (NPY) Modulates Experimental Colitis

**DOI:** 10.1371/journal.pone.0003304

**Published:** 2008-10-01

**Authors:** Bindu Chandrasekharan, Vanitha Bala, Vasantha L. Kolachala, Matam Vijay-Kumar, Dean Jones, Andrew T. Gewirtz, Shanthi V. Sitaraman, Shanthi Srinivasan

**Affiliations:** 1 Division of Digestive Diseases, Emory University, Atlanta, Georgia, United States of America; 2 Department of Pathology and Laboratory Medicine, Emory University, Atlanta, Georgia, United States of America; 3 Division of Pulmonary Medicine, Emory University, Atlanta, Georgia, United States of America; University of Iowa, United States of America

## Abstract

**Background:**

Neurogenic inflammation plays a major role in the pathogenesis of inflammatory bowel disease (IBD). We examined the role of neuropeptide Y (NPY) and neuronal nitric oxide synthase (nNOS) in modulating colitis.

**Methods:**

Colitis was induced by administration of dextran sodium sulphate (3% DSS) or streptomycin pre-treated *Salmonella typhimurium* (*S.T.*) in wild type (WT) and NPY (*NPY^−/−^*) knockout mice. Colitis was assessed by clinical score, histological score and myeloperoxidase activity. NPY and nNOS expression was assessed by immunostaining. Oxidative stress was assessed by measuring catalase activity, glutathione and nitrite levels. Colonic motility was assessed by isometric muscle recording in WT and DSS-treated mice.

**Results:**

DSS/*S.T.* induced an increase in enteric neuronal NPY and nNOS expression in WT mice. WT mice were more susceptible to inflammation compared to *NPY^−/−^* as indicated by higher clinical & histological scores, and myeloperoxidase (MPO) activity (p<0.01). DSS-WT mice had increased nitrite, decreased glutathione (GSH) levels and increased catalase activity indicating more oxidative stress. The lower histological scores, MPO and chemokine KC in *S.T.*-treated *nNOS^−/−^* and *NPY^−/−^/nNOS^−/−^* mice supported the finding that loss of NPY-induced nNOS attenuated inflammation. The inflammation resulted in chronic impairment of colonic motility in DSS-WT mice. NPY –treated rat enteric neurons *in vitro* exhibited increased nitrite and TNF-α production.

**Conclusions:**

NPY mediated increase in nNOS is a determinant of oxidative stress and subsequent inflammation. Our study highlights the role of neuronal NPY and nNOS as mediators of inflammatory processes in IBD.

## Introduction

Recently neural immune regulation has been identified to play a major role in the pathogenesis of inflammatory bowel disease (IBD) [1], [2], [3]. Changes in the enteric nervous system (ENS) observed during IBD include, modifications in morphology (neuronal hyperplasia and large internodal fiber tracts), neuromediator content of enteric neurons and changes in the function of enteric neurons (impaired release of neuromediators) [4]. These changes in enteric neurons are likely to play a role in alterations in motility and secretory functions of the gastrointestinal tract [5]. Neurotrophins such as Nerve Growth Factor (NGF) increase in inflamed tissues and NGF have been shown to be increased in patients with Crohn's disease (CD) and ulcerative colitis (UC) [6]. The mechanisms by which dysregulated immune response initiates and perpetuates inflammatory intestinal injury are not entirely known. There is evidence that gastrointestinal inflammation is related to an imbalance in the function of peptidergic neurons, including Substance P (SP), Vasoactive Intestinal Peptide (VIP) and NPY [7], [8]. Neuropeptides like SP are up regulated in DSS model of experimental colitis, and there is increased expression of SP binding sites in UC and CD [9].

The NPY family of neuropeptides, including NPY, peptide YY (PYY), and pancreatic polypeptide (PP), has been shown to elicit diverse biological functions including hypothalamic control of food intake, anxiety and sedation. These polypeptides are coupled to heptahelical G-protein coupled receptors. NPY receptor co localizes with NPY-producing nerve cells and is widely distributed in the enteric and the central nervous system (CNS).

We have previously demonstrated that neuropeptide Y (NPY) increases the number of nNOS (neuronal nitric oxide synthase) containing neurons *in vitro* and in NPY knock out (*NPY^−/−^*) mice there is a reduction in nNOS neurons [10]. The nNOS produces nitric oxide (NO), a major Non-adrenergic Non-cholinergic (NANC) neurotransmitter, which mediates relaxation responses of smooth muscles in the gastrointestinal (GI) tract. nNOS has been identified to play a role in a variety of enteric neuropathies like Crohn's disease, ulcerative colitis, Chagas disease, diabetic gastroparesis, achalasia and pyloric stenosis [11].

The role and expression of NPY from enteric neurons in intestinal inflammation are not known. In the present study we analyzed the role of NPY in modulating inflammation associated with DSS and *Salmonella* models of colitis. We hypothesized that an increase NPY leads to an increase in nNOS neurons, resulting in increased oxidative stress, thereby aggravating colonic inflammation. Studies were performed in WT, *NPY^−/−^*, *nNOS^−/−^* as well as *NPY^−/−^/nNOS^−/−^* double knock out mice models to understand the effects of absence of either NPY or nNOS in colonic inflammation.

## Materials and Methods

### Reagents and Antibodies

Tyramide signal amplification kit (TSA-Cy-3 and TSA-FITC)- NEN Life Science Products, Wellesley, MA; dextran sodium sulfate (DSS, molecular weight, 50000; Lot no 3414 H) - MP Biomedicals, Aurora, OH; NPY - BACHEM, King of Prussia, PA; L-NAME -Cayman Chem, Ann Arbor, MI; Omniscript Reverse Transcription kit -Qiagen, Valencia, CA; NPY primers -IDT, Taq DNA polymerase and Greiss assay kit -Promega, Madison, WI. All chemicals not included above were from Sigma, St.Louis, MO. The real time PCR was performed using Fast SYBR® Green Master Mix from Applied Biosystems, Foster city, CA. The antibodies to NPY, nNOS and peripherin (Chemicon International, Temecula, CA), Donkey anti-rabbit biotin and peroxidase-conjugated streptavidin from Jackson Immuno Research Laboratories, Inc, West grove, PA.

### Mice

All animal studies were approved by the Emory University Institutional Animal Care and Use Committee (IACUC). WT control (Stock No. 002448, 129 S3/SvImJ) *NPY^−/−^* mice (Stock No. 004545, strain 129S-Npy^tm1Rpa^/J) [12] and *nNOS knock out mice* (*nNOS^−/−^*) (Stock No 002633, strain B6; 129 S4-*Nos1^tm1Plh^*/J) were obtained from The Jackson Laboratory, Bar Harbor, ME. In *nNOS knock out mice (nNOS^−/−^)* a neomycin cassette replaced exon 1 which encodes the initiation site and amino acids 1–159 of the neuronal nitric oxide synthase (nNOS 1) protein.

### Generation of *NPY^−/−^/nNOS^−/−^* Double knock out mice

*NPY^−/−^* mice and *nNOS^−/−^* mice were obtained from The Jackson Laboratory were crossed in our animal facility to obtain the F1 heterozygotes, which were then crossed for obtaining the double knockouts. The corresponding double positive mice from breeding were used as the WT control in the experiments. The genotype of all the mice were verified prior to experiments by RT-PCR of mouse tail using the RED-Amp Kit (Sigma).

### Induction of DSS colitis

Colitis was induced in eight week old WT and *NPY^−/−^*age and sex matched male and female WT and *NPY^−/−^* littermates by oral administration of 3% (w/v) DSS in tap water *ad libitum* for 6 days. Age-matched male and female WT and *NPY^−/−^* mice receiving tap water served as controls. Mice were evaluated for clinical symptoms of colitis. The animals were sacrificed on day 6 and tissues were processed for analyses.

### Induction of *Salmonella typhimurium*-induced gastroenteritis

Gut-restricted *S. typhimurium (S.T.)*, (strain SL3201) infection was induced after pre-treatment with streptomycin according to Barthel et al [13] in eight week old WT , *NPY^−/−^*, *nNOS^−/−^* and *NPY^−/−^/nNOS^−/−^* double knock out mice. After 48 hrs post infection, cecum and colon samples were collected. Gross change in cecal morphology in control and *S.T* infected group was evaluated.

### Clinical Score

Body weight, stool consistency, presence of occult or gross blood in feces by guaiac test (Hemoccult Sensa, Beckman Coulter, Fullerson, CA) were assessed daily for each animal in DSS colitis and clinical scoring done as per Cooper et al [14]. Mice were sacrificed by carbon dioxide on day 6. The length of colon was also measured.

### Histological score

Distal colon sections were stained with hematoxylin and eosin (H/E) [15] and damage was scored as per Cooper *et al* [14].

### Myeloperoxidase Assay

Distal colons from DSS treated mice (day 6) and cecum from *S.T.* -treated mice (48 h) were snap-frozen in liquid nitrogen, and myeloperoxidase activity was measured [16] and expressed per µg of protein.

### KC Assay

WT, *NOS^−/−^* and *NPY^−/−^/NOS^−/−^* mice were bled at 48 hours after *S.T.* infection through the retro-orbital plexus. The serum was isolated by centrifugation and levels of murine neutrophil chemoattractant KC assayed using the mouse Duoset ELISA kits [17] (R&D Systems).

### Reverse Transcription and Real-Time Polymerase Chain Reaction

Total RNA was isolated from distal colon (RNeasy Mini Kit , Qiagen) from WT/*NPY^−/−^* mice treated with water or DSS or *S.T.* The reaction mixtures were then set up with cDNA and primers for NPY [18], [19]. The amplification products were visualized on a 1.5% agarose gel. NPY expression was normalized to 18S rRNA. The real-time data was analyzed using BioRad MyiQ Optical System software 2.0.

### Immunofluorescence Studies

Distal colon from individual WT and *NPY^−/−^* mice treated with water or DSS were processed and stained with anti-NPY or anti-*nNOS* antibodies (1∶1000) and Peripherin was used as a neuronal marker (1∶5000), as described previously [10].

### Confocal Microscopy

Zeiss LSM410 microscope was used and green fluorescence was excited at 488 nm and detected with a 515–540 nm band-pass filter. Red fluorescence was excited at 543 nm and detected with a 570 nm long-pass filter. The intensity of staining in the ganglion was assessed using the Metamorph Imaging system, Molecular Devices, Sunnyvale, CA. At least 8 ganglia were measured to get the average nNOS intensity. The intensity assessment was done by a researcher blinded to the treatment groups.

### Catalase Activity

The distal colon segments from control, DSS- WT and DSS-*NPY^−/^* ^−^ mice were collected and catalase activity was determined by spectrophotometric method [15], [20].

### Nitrite estimation in the colon

Distal colon collected from control and DSS –treated mice (WT and *NPY^−/−^*) was homogenized in phosphate buffered saline and supernatant was used for the assay using Greiss assay kit.

### Glutathione assay

Colonic tissue (50 mg) was processed for reduced glutathione (GSH) analysis by HPLC [21].

### Nitric oxide and TNF- α production from neuronal cultures

Enteric neurons isolated from E14 rat embryos as previously described [19] were exposed to NPY (1 µM) for 24 h. The supernatants collected at 24 h were used for nitrite analysis (Greiss assay) and TNF- α (ELISA kit BioSource, Invitrogen, CA).

### Assessment of colonic motility by Isometric muscle recording with electrical field stimulation (EFS)

The relaxation responses from the longitudinal muscle strips containing the myenteric plexi from the proximal colon of WT, *NPY^−/−^*, *NOS^−/−^* and *NPY^−/−^/nNOS^−/−^* mice were assessed as described earlier [11]. The responses from DSS- treated WT mice at 0, 2 and 5 weeks after withdrawal of DSS were also determined. For quantitative determination of responses, at least 3 animals were used in each group and the average response obtained.

### Statistical analysis

Data were analyzed by t-test or One-way ANOVA with Tukey's post test, using GraphPad Prism version 3.00 for Windows, GraphPad Software, San- Diego California, USA. Results were considered significant at *P*<0.05.

## Results

### NPY expression is up regulated in enteric neurons during experimental colitis in WT mice

**Figure 1:**
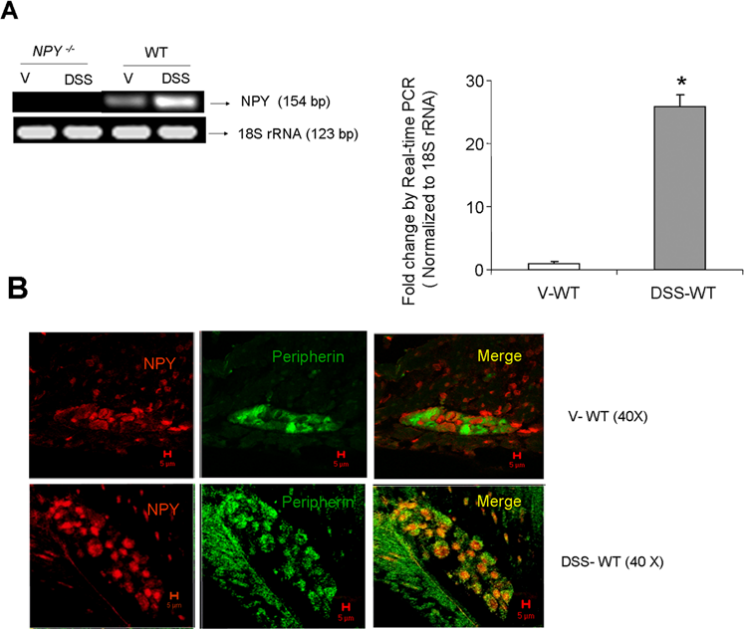
DSS induces NPY expression in the enteric ganglia. (A) RNA was isolated from WT and *NPY* ^−/−^ mice given water or 3% DSS and amplified for NPY and 18S rRNA (loading control). The increase in NPY mRNA expression as assessed by real-time PCR is graphically represented as fold change compared to control. Each bar represents mean±S.E; n = 4, Significant difference from all other groups represented as * p<0.05, ** p<0.01 (B) Confocal images of single enteric ganglion from control and DSS- treated WT mice assessed for peripherin (green) and NPY (red) are shown. Magnification 40X, Scale bar 5 µm.

Reverse-transcription PCR indicated an increase in NPY expression after DSS and *Salmonella* exposure (Figure 1A and Figure 3A). Further experiments by Real-time PCR indicated a significant increase in NPY mRNA (25.77±2.67, p<0.05) after two days of DSS exposure in WT mice (graphically represented in Figure 1A as fold change compared to control), and also after *S.T.* treatment (5.96±0.7, p<0.01, Figure 3A, graphically represented as fold change compared to control). Immunostaining revealed increased NPY expression in the enteric ganglia of DSS (Figure 1B) and *S.T.*-treated (Figure 3B) animals compared to controls. Thus both DSS and *S.T.* treatment induced an increase in neuronal NPY expression.

### *NPY^−/−^* mice are resistant to the development of Dextran sodium sulfate and *Salmonella*- induced colitis

**Figure 2:**
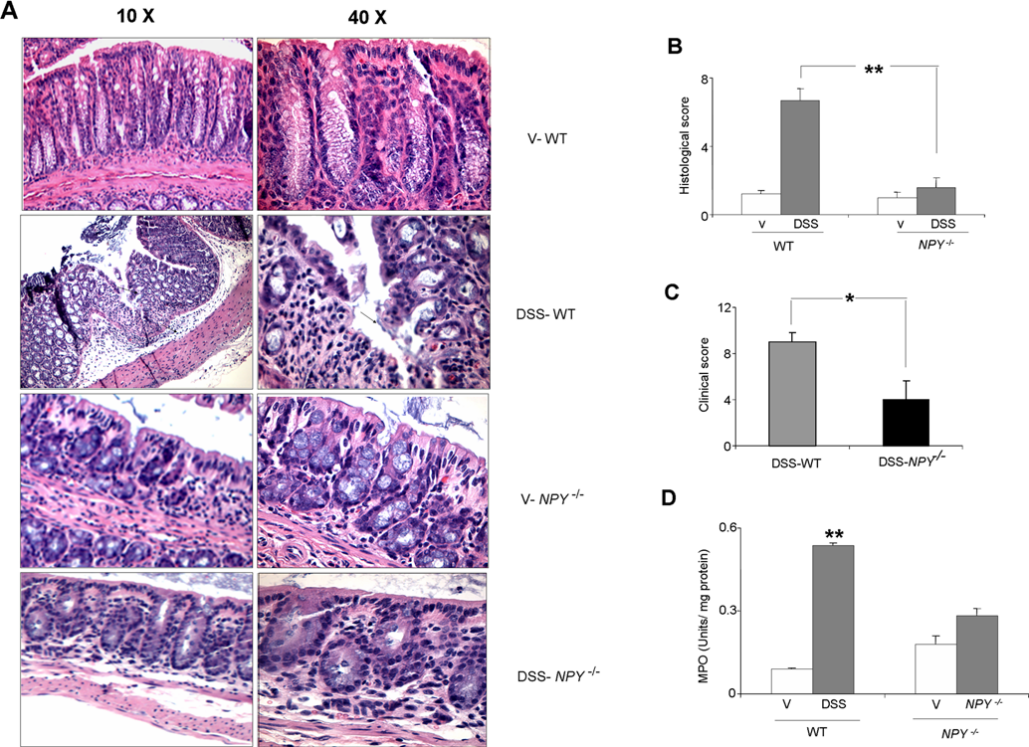
Lack of NPY inhibits DSS-induced inflammation. Mice were randomized into 4 groups: WT water or DSS (3%) and *NPY* ^−/−^ water or 3% DSS and sacrificed on day 6. (A) Representative hematoxylin eosin (H/E) stained sections of colon from each group are shown. Arrows represent neutrophil infiltration and crypt damage. Magnification 10X and 40X. Disease severity was assessed as described in the Materials and Methods and expressed in terms of (B) Histological score (C) Clinical score and (D) Myeloperoxidase activity. Each bar represents mean±S.E; n = 4, Significant difference from all other groups represented as * p<0.05, ** p<0.01.

Since DSS/*S.T.* induced NPY expression in the enteric ganglia, we next assessed the effects of lack of NPY on inflammation. The *NPY^−/−^* mice were resistant to DSS colitis as assessed by histological score, clinical score and blood in stool (Figure 2, B–D). DSS-WT mice had a significant (10.91±1.5%, p<0.05) reduction in body weight and increased blood loss as compared with controls. In contrast *NPY^−/−^* mice exhibited less inflammation with an overall clinical score of 4±0.7 compared to a score of 9±0.5 in DSS-WT mice (p<0.01, Figure 2C). Reduction of colon length, which is used as a parameter of inflammation, correlated with the clinical scores [22]. The colon length of DSS-WT mice was significantly less (5.0±0.08, p<0.05) as compared to the controls (6.0±0.2). However in *NPY^−/−^* group there was no significant differences in colon length between DSS and control mice.

DSS-induced colitis is featured by colonic inflammation manifested by crypt damage, mucosal destruction, epithelial erosions and infiltration of inflammatory cells into the mucosa. Tissues from DSS treated WT and *NPY^−/−^* mice were examined histologically and compared with the controls. The histological data supported the observations based on clinical analysis and confirmed the protective role of NPY deletion towards the development of colitis (Figure 2 A). Distal colonic segments collected from DSS (Figure 2A) and *Salmonella* treated (Figure 4A) WT mice showed visible signs of colonic inflammation and tissue damage. There was extensive crypt damage, epithelial erosion, ulcer formation and infiltration of neutrophils into the lamina propria and muscularis of colonic sections. In contrast histological analysis of colonic sections from *NPY^−/−^* mice showed significantly reduced histological inflammation, and there was reduced mucosal damage and less infiltration. The histological score (Figure 2B) on DSS treatment was significantly lower in *NPY^−/−^* mice (1.6±0.55) compared to that in WT mice (6.69±0.69). The histological score in *S.T.* treated WT mice was significantly higher (10±0.5) when compared to the *NPY^−/−^* mice (5±0.8) indicating a better resistance (Figure 4D) associated with deletion of NPY. The *S.T.* infection is characterized by shrinkage of cecum (Figure 4B) and we found that WT mice suffered from 43.8% loss of cecum weight as compared to 2.4% in *NPY^−/−^* mice (Figure 4C).

**Figure 3:**
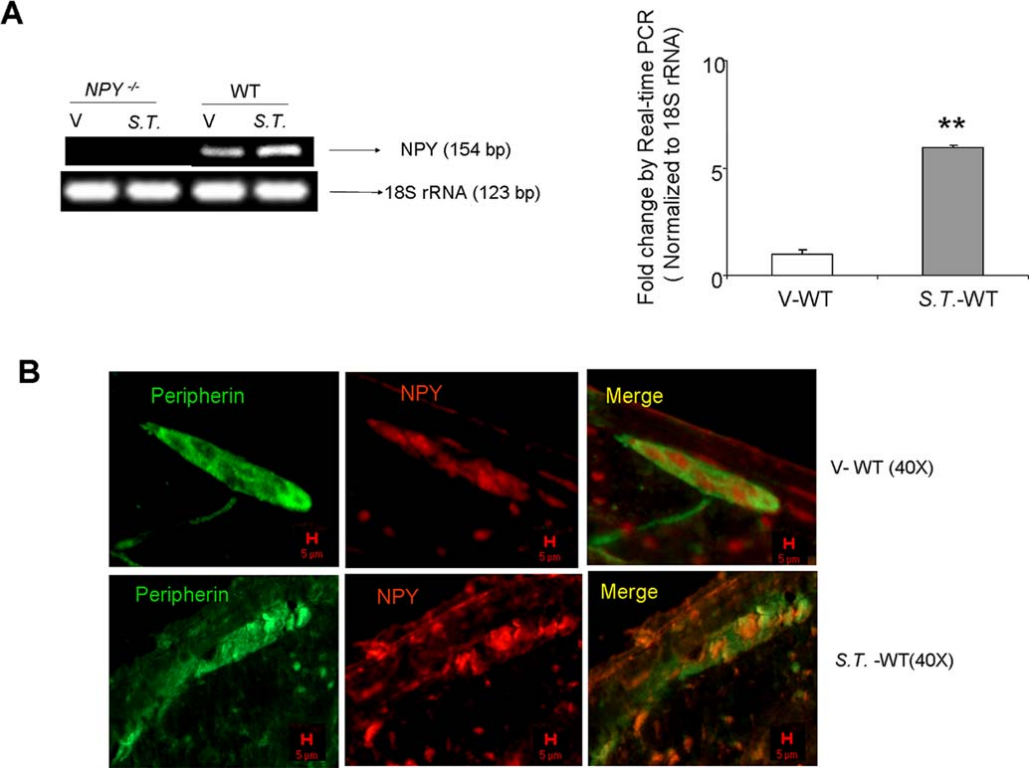
*S.T.* induces NPY expression in the enteric ganglia. (A)RNA was isolated from WT and *NPY* ^−/−^ mice given water or *S.T.* (48 hrs), and amplified for NPY and 18 S RNA (loading control). The increase in NPY mRNA expression as assessed by real-time PCR is graphically represented as fold change compared to control. Each bar represents mean±S.E; n = 4, Significant difference from all other groups represented as * p<0.05, ** p<0.01 (B) Confocal images of single enteric ganglion from control and *S.T.*- treated WT mice assessed for peripherin (green) and NPY (red) are shown. Magnification 40X, Scale bar 5 µm.

**Figure 4:**
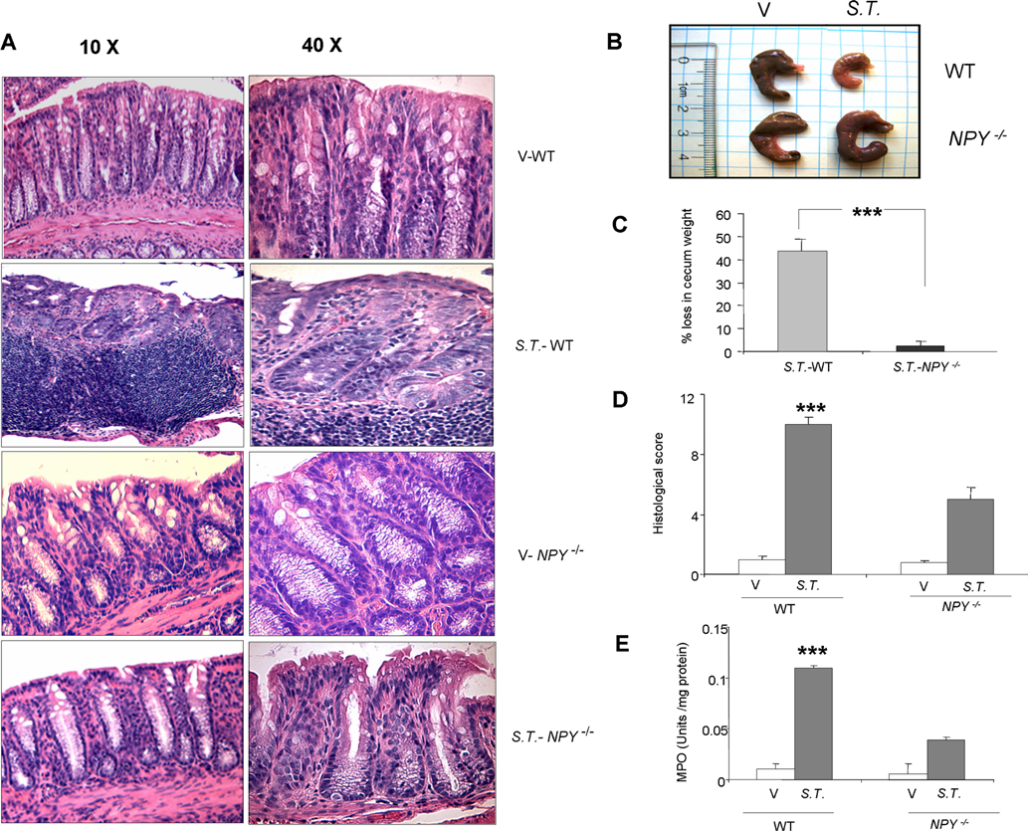
*NPY* ^−/−^ mice are resistant to *S.T.*-induced colitis. (A) Representative sections of hematoxylin eosin (H/E) stained sections of colon from WT and *NPY* ^−/−^ mice given water/*S.T.*. Arrows represent neutrophil infiltration and crypt damage. Magnification 10X and 40X. (B) Morphology of cecum (C) Percent cecum shrinkage in WT mice and *NPY* ^−/−^ mice. Disease severity was assessed as described in the Materials and Methods section in terms of (D) Histological score and (E) Myeloperoxidase activity. Each bar represents mean±S.E; n = 4, Significant difference from all other groups represented as *** p<0.001.

Neutrophil infiltration into the injured tissue is a characteristic feature of colitis. To validate the histological findings we assessed the myeloperoxidase activity (MPO), an index of neutrophil infiltration, in the colon (DSS treated) and cecum (*S.T.* treated) of WT and *NPY^−/−^* mice. Compared to WT control, the mice receiving DSS had significantly higher (p<0.01) MPO activity (Figure 2D). In contrast, MPO activity in DSS-treated *NPY^−/−^* mice was 50% less than DSS-WT mice. *S.T.* infection also resulted in higher MPO activity in WT mice as compared to the *NPY^−/−^* mice (Figure 4E). These data indicate that deletion of NPY attenuated clinical as well as histological characteristics associated with DSS and *salmonella*-induced colitis.

### NPY induces nNOS neurons and enhances nitrite levels

**Figure 5:**
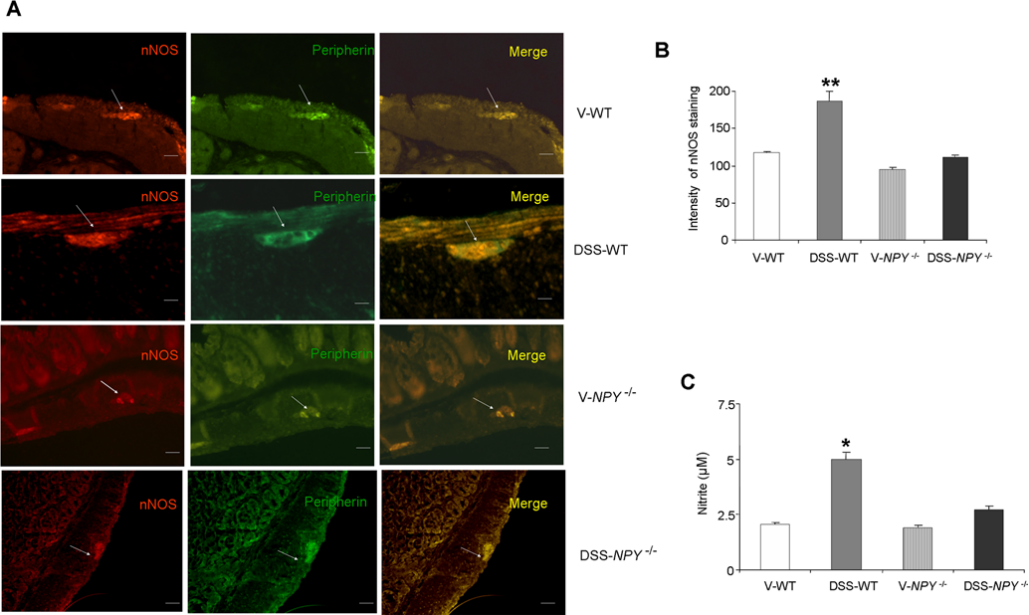
Neuronal NOS (nNOS) expression and nitrite levels are increased in DSS-treated mice. (A) Distal colons of WT and *NPY* ^−/−^ mice treated with water and DSS (3%), were fixed in formalin and processed for nNOS immunostaining as described in methods section, Magnification 20X, Scale bar 20 µm. (B) Graphical representation of relative intensity of nNOS staining as determined by Metamorph program (C) Nitrite levels, an index of nitric oxide production, were measured by Greiss assay as described in Materials and methods section. Each bar represents mean±S.E; n = 4, Significant difference from all other groups represented as * p<0.05, **p<0.01.

We have previously demonstrated that *NPY^−/−^* mice have a reduced number of nNOS enteric neurons [10]. To assess if DSS induced NPY can modulate nNOS, we assessed the expression of nNOS in enteric ganglia by immunofluorescence. DSS induced a significant increase in nNOS (Figure 5A) expression in the enteric ganglia of WT mice as compared to *NPY^−/−^* mice as graphically represented in Figure 5B. This increased expression of nNOS in WT mice resulted in higher levels of nitric oxide production as revealed from higher nitrite levels (p<0.05) as compared to *NPY^−/−^* mice (Figure 5C). The *NPY^−/−^* mice lack NPY and hence have less nNOS, and this can contribute to decreased nitric oxide production and nitrite levels in response to DSS.

### *NPY^−/−^* mice have decreased oxidative stress

**Figure 6:**
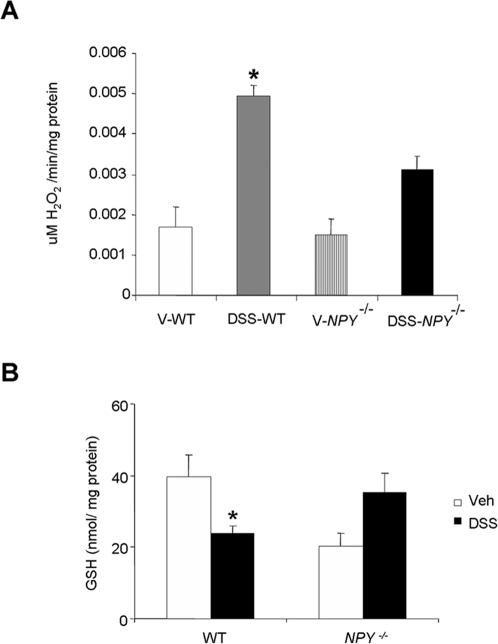
Oxidative stress is significantly reduced in *NPY* ^−/−^ mice. Distal colons were snap-frozen in liquid nitrogen for assessment of oxidative stress. (A) Catalase activity was measured spectrophotometrically as an index of oxidative stress as described in Materials and methods section, and (B) Colonic GSH levels in vehicle (water-treated) and DSS-treated WT and *NPY* ^−/−^ mice as measured by HPLC. Each bar represents mean±S.E; n = 4, Significant difference with respect to other groups represented as * p<0.05.

We next examined if the DSS-induced NPY expression and subsequent nNOS expression resulted in increased oxidative stress. The catalase activity, an index of peroxide stress, was significantly higher (p<0.05) in colonic tissues of DSS- WT mice when compared to *NPY^−/−^* mice (Figure 6A). The levels of reduced glutathione (GSH) was significantly less in WT mice exposed to DSS, further pointing to increased oxidative stress, where as GSH levels were higher in DSS-*NPY^−/−^* mice (Figure 6B).

### *nNOS^−/^* ^−^ and *NPY^−/−^ nNOS^−/−^* mice are resistant to the development of *Salmonella*- induced colitis

**Figure 7:**
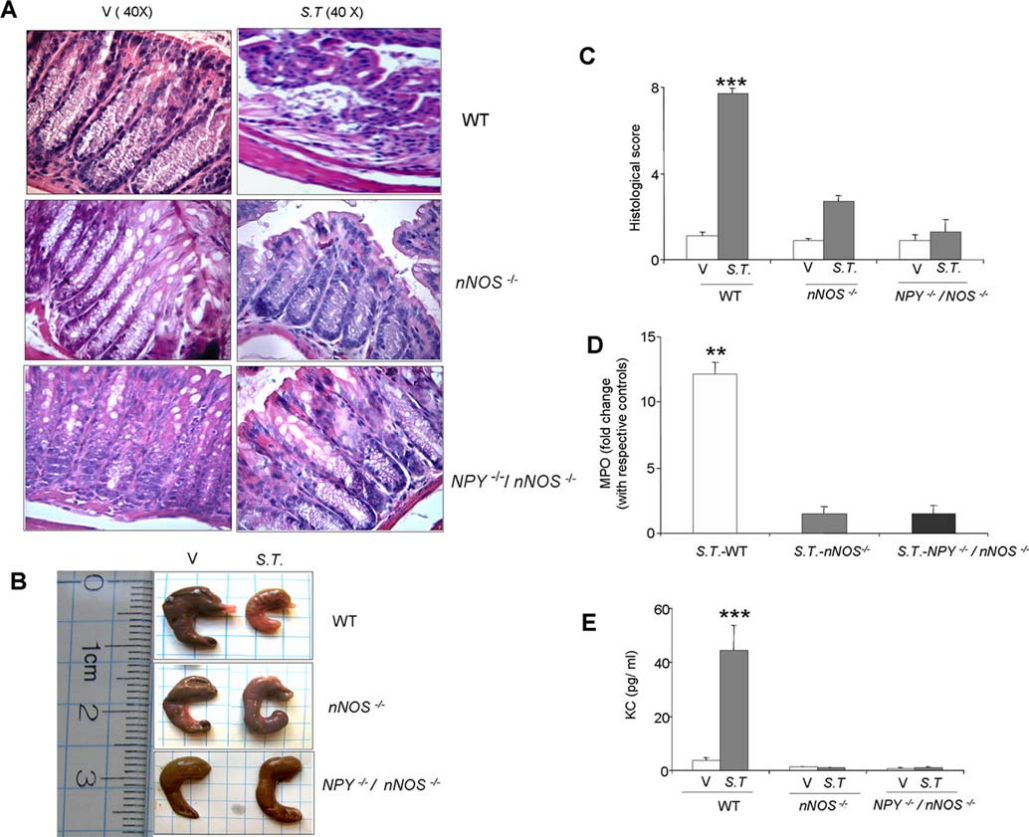
*nNOS* ^−/−^ and *NPY* ^−/−^/*nNOS* ^−/−^ mice resist *S.T.* colitis. Distal colon was isolated from mice given water or *S.T.* (48 hrs) (A) Representative hematoxylin eosin stained sections of colon from each group are shown. Magnification 40X, Scale bar 100 µm. Disease severity was assessed as described in the Materials and Methods section in terms of (B) Morphology of cecum (C) Histological score (D) MPO activity and (E) KC levels in the serum at 48 hrs of *S.T.* infection. Each bar represents mean±S.E, n = 4, Significant difference with respect to other groups represented as *** p<0.001.

In order to validate the observation that NPY mediated increase in nNOS is responsible for increased oxidative stress and subsequent damage in colitis; we induced *S.T.* colitis in *nNOS^−/−^* and *NPY^−/−^/nNOS^−/−^* mice. These results are represented in Figure 7 (A–E). Both *nNOS^−/−^* and *NPY^−/−^/nNOS^−/−^* mice exhibited less inflammation as evident from histological damage (Figure 7A), less cecum shrinkage (Figure 7B), decreased histological score (Figure 7C) and MPO activity (Figure 7D). KC is a chemokine in mice that is capable of recruiting and activating neutrophils. It is the human homolog of IL-8 in mice, and hence is a useful marker to assess inflammation. We found that KC levels were much less in *nNOS^−/−^* and *NPY^−/−^/nNOS^−/−^* mice (Figure 7E) as compared to the respective wild type controls. This suggested that NPY-mediated damage in colitis is through nNOS.

#### Colonic relaxation was chronically impaired in WT mice after DSS treatment

**Figure 8:**
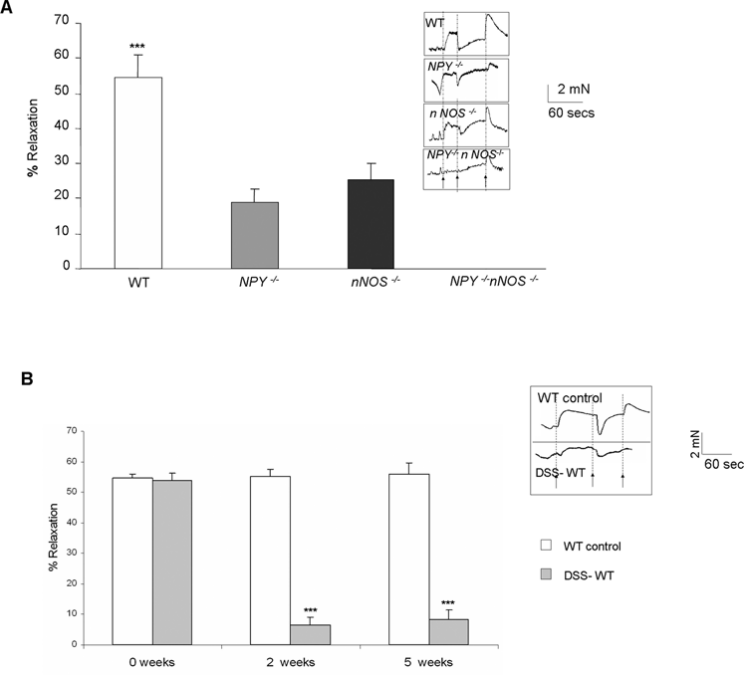
Colonic motility in WT, *NPY* ^−/−^, *nNOS* ^−/−^ and *NPY* ^−/−^/*nNOS* ^−/−^ mice. EFS-induced relaxation of longitudinal muscle strips was assessed as described in Materials & Methods. Transmural EFS-induced relaxation (24 V, 8 Hz, 5 milliseconds, 60 seconds) of the proximal colon was assessed in mice colons pre-contracted with 5-HT in presence of atropine and guanethidine. Representative tracings of NANC relaxation in (A) WT, *NPY* ^−/−^, *nNOS* ^−/−^ and *NPY* ^−/−^/*nNOS* ^−/−^ vehicle mice, and (B) in DSS-treated WT mice at 2 and 5 weeks after DSS treatment are presented and also graphically represented. The y axis represents force in mN, and the x axis represents time with arrows representing 5-HT, EFS-on and EFS-off respectively. Percentage relaxation was calculated by determining the difference between the maximal force generated at baseline and the minimum force following electrical stimulation and expressing this as a percentage of the force generated at baseline. Bar graphs represent the percent relaxation responses with respect to 5-HT-induced contraction. Each bar represents mean±S.E; n = 3 for each group, Significant difference from all other groups represented as ***p<0.001.

Among the various inhibitory neurotransmitters, neuronal nitric oxide (NO) generated by nNOS contributes mainly to NANC relaxation. The NANC relaxation in the longitudinal muscle strips (containing the myenteric plexus) from the proximal colon of WT, *NPY^−/−^*, *nNOS^−/−^* and *NPY^−/−^/NOS^−/−^* control mice is represented in Figure 8A. The WT control mice that harbor both NPY and nNOS exhibited maximum relaxation response (54.6±6.26%). As previously demonstrated [10], *NPY^−/−^* mice have reduced relaxation responses (18.78±4.05%) due to a reduction in nNOS neurons. We found that *nNOS^−/−^* mice (in spite of having NPY) also exhibited decreased relaxation response (25.27±4.63%). There was only minimal relaxation in the *NPY^−/−^ NOS^−/−^* double knock out mice. This indicates that majority of relaxation response is mediated by NPY and nNOS. This data confirmed the role of NPY and nNOS as the major determinants of NO- mediated relaxation. In the DSS-treated WT mice, inflammation results in destruction of neurons and loss of relaxation responses. The impairment in relaxation was persistent even 5 weeks after DSS treatment indicating more inflammation and damage to neurons (Figure 8B).

### NPY-treated enteric neurons exhibit increased nitrite and TNF-α production

**Figure 9:**
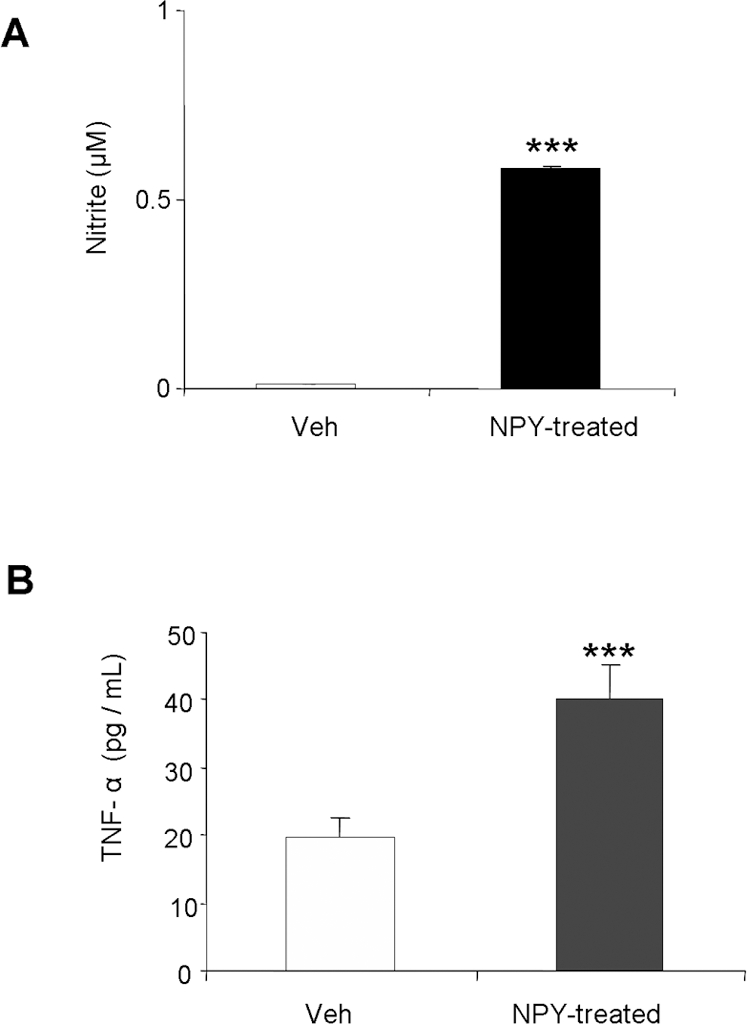
NPY increases nitric oxide production and TNF-α release from enteric neurons *in vitro*. Enteric neuronal cells were cultured in DMEM in presence or absence of NPY (1uM). After 24 h, media was collected from each well and analyzed for (A) nitrite and (B) TNF-α release as detailed in methods section. Each bar represents mean±S.E, n = 4, Significant difference from all other groups represented as * p<0.05, ***p<0.001.

We have previously shown that NPY can increase nNOS neurons *in vitro* [10]. In the present study we observed that NPY can induce nitric oxide production and TNF-α release from enteric neuronal cultures. There was increased nitric oxide production as seen from significantly higher nitrite (0.585±0.002 µM, p<0.001) in NPY-treated enteric neuronal cultures compared to untreated controls (Figure 9 A). Similarly, TNF-α release (pg/mL of media) was also significantly higher from NPY-treated neuronal cultures (40.12±7.7) compared to untreated controls (19.59±2.41) (Figure 9 B). These findings indicate that NPY contributes to increased nitrosative stress and enhances release of inflammatory mediators like TNF-α. Thus, NPY-containing neurons can make a significant contribution during an inflammatory process.

## Discussion

The enteric nervous system has been shown to play an important role in the pathophysiology of inflammatory bowel disease [23]. The present study highlights the role of enteric neurons as modulators of inflammatory processes in IBD. NPY is produced by enteric neurons as well as the central nervous system (CNS). Of the total neurons in the mice colon, NPY containing neurons represent 50% of the total neurons in myenteric plexi and less than 5% neurons in the sub mucosal plexi [24]. The role of NPY from immune cells has been described in inflammation. Our study demonstrates increased colonic expression of NPY in DSS and *S.T.* colitis. Thus, NPY is an inducible gene in enteric neurons which can promote inflammation.

To investigate the role and involvement of NPY in the development of chemical and infectious colitis, mice with targeted disruption of NPY gene, and WT mice were evaluated for clinical and histopathologic manifestations of colitis. DSS-induced colitis is a well established preclinical model to understand the pathophysiology of intestinal inflammation as it bears a close resemblance to human colitis [25]. Our study demonstrates that WT mice exposed to DSS shows significant weight loss, increased fecal blood and neutrophil infiltration, crypt damage and epithelial erosions. Unlike WT mice, *NPY^−/−^* mice exhibited significantly lower clinical and histological manifestations in response to DSS. Similar histological findings indicating more inflammation was also noted in *S.T* -treated WT mice, as characterized by cecum shrinkage, crypt damage and ulceration when compared to *NPY^−/−^* mice.

Polymorphonuclear leukocyte migration across the epithelium is considered as a key feature in active inflammatory diseases like chronic ulcerative colitis (CUC) and IBD. There was significant accumulation of neutrophils in the colonic mucosa of WT mice, however there was only minimal inflammatory infiltrates in knock out mice, suggesting the role of NPY in neutrophil recruitment. It has been demonstrated that activation of sympathetic neuro transmitters like NPY and epinephrine mobilizes blood leucocytes [26]. NPY has also been demonstrated to prime polymorphonuclear (PMN) oxidative metabolism [27]. Similarly, we also noted increased MPO activity in WT mice treated with DSS/*S.T.* as compared to the *NPY^−/−^* counterparts.

Our studies demonstrate a role for the neuronal NOS, along with NPY, in the pathogenesis of colitis. Our laboratory has previously shown that NPY can increase nNOS neurons *in vitro* [10], and that *NPY^−/−^* mice have reduced number of nNOS neurons. In the current study we observed that DSS induces NPY expression and up regulation of nNOS. The role of nitric oxide synthases and nitric oxide in experimental colitis has been controversial. Several studies have shown that NOS inhibitors like L-NAME has been able to attenuate [28], [29] or exacerbate [30] TNBS-induced colitis. We observed a significant upregulation of nNOS (by immunostaining) on the enteric ganglia in WT mice compared to *NPY^−/−^* mice. We believe that the increased nitrite noted in colitis is due to the increased nNOS. Our *in vitro* studies now demonstrate that enteric neuronal cultures treated with NPY had increased nitrite levels, which indicates increased nitric oxide production.

Colonic oxidative stress is a hallmark of IBD and colonic nitrite levels serve as a sensitive marker of disease activity in colitis [31] . It has been demonstrated that increase in nitric oxide in colon in ulcerative colitis is from myenteric neurons and not epithelium or endothelium [32]. There was further evidence of increased oxidative stress in the DSS-treated WT mice as seen by the significant increase in catalase activity and reduced GSH levels. Inflammatory cells may also contribute to elevated nitrite levels and catalase activity due to increased neutrophil infiltration (as evidenced by increased myeloperoxidase activity) that occurs in presence of NPY.

In order to validate the observation that NPY-mediated increase in nNOS is responsible for increased oxidative stress and subsequent damage in colitis, we used *S.T.* model to study the effect of the absence of nNOS as well as absence of both NPY and NOS in inflammation. We observed that inflammation was significantly reduced in *nNOS^−/−^* mice as well as *NPY^−/−^/nNOS^−/−^*double knockouts. The presence of NPY in the *nNOS^−/−^* could not help restore the *S.T.* induced inflammatory response. Isometric muscle recording data support reduced relaxation response in *NPY^−/−^*, *nNOS^−/−^* and *NPY^−/−^/nNOS^−/−^* mice. This supports our hypothesis that NPY-nNOS induced nitric oxide is a major determinant of oxidative stress -induced damage in colitis.

It is known that post ganglionic sympathetic nerve fibers terminate in lymphoid organs and secrete NPY which regulates multiple physiological processes in the body [33]. NPY can also activate macrophages and immune cells, Th1 responses, and stimulate the production of various cytokines [34], [35]. TNF *–α* production from the spinal cord neurons has been demonstrated in inflammatory central nervous system diseases like experimental allergic encephalomyelitis [36]. Our study has demonstrated that NPY has the capacity to stimulate TNF-α production from enteric neurons in culture. This observation highlights the fact that enteric neurons are additional sources of cytokines during inflammatory processes and that NPY-containing neurons make a significant contribution during an inflammatory process.

The increased severity of inflammation in the WT mice was also noted by functional impairment of neuronally-mediated muscle contractions and relaxations even after 5 weeks of DSS withdrawal. This indicates that neuronal damage secondary to inflammation can lead to long term gastrointestinal motility problems.

In summary, our study demonstrates that NPY from enteric neurons is up regulated during colitis and it can regulate the expression of nitric oxide synthase and subsequent nitric oxide production. Possible mechanisms of NPY-mediated inflammation are via increased oxidative stress and cytokine release. The inflammation in turn results in chronic impairment of GI motility. Taken together our data reveal that enteric neurons producing NPY play an important role in modulating inflammation in colitis. NPY-dependent mechanisms participate in the adaptive changes of neurons in response to injury/inflammation. We show that the enteric nervous system not only participates in the pathophysiology of inflammation, but also is affected as a consequence of inflammation. Persistence of the changes in enteric neurons in the recovery phase can explain some of the motility disturbances seen in patients with inflammatory bowel disease even in the absence of inflammation. Hence targeting this neuropeptide may have a therapeutic value in managing inflammatory bowel disease.
